# The Occurrence of Health Symptoms in General Practice Before and After the Explantation of Cosmetic Breast Implants

**DOI:** 10.1093/asj/sjaf030

**Published:** 2025-02-19

**Authors:** Annemiek S Lieffering, Marc A M Mureau, Juliëtte E Hommes, Lotte Ramerman, Hinne A Rakhorst, René R W J van der Hulst, Robert A Verheij

## Abstract

**Background:**

Explantation of breast implants is increasingly performed in response to concerns about breast implant illness (BII), an array of various health symptoms. However, the benefits of explantation remain unclear because of methodological limitations in previous studies.

**Objectives:**

To examine the occurrence of health symptoms before and after explantation of cosmetic breast implants.

**Methods:**

This is a retrospective cohort study linking data from the Dutch Breast Implant Registry and Nivel Primary Care Database. The study included 217 cosmetic explantation patients, control groups of 228 cosmetic replacement patients, and 433 female patients without breast implants (nonrecipients). BII-related health symptoms presented in general practice were compared between groups 1 year before and after explantation. Outcomes included any symptom, ≥2 symptoms, ≥3 symptoms, ≥3 consultations, and substantial symptoms (≥3 symptoms with ≥2 consultations for 2 symptoms).

**Results:**

The likelihood of ≥3 symptoms, ≥3 consultations, and substantial symptoms reduced significantly after explantation (odds ratio [OR] ≥3 symptoms 0.26, 95% CI [0.08-0.85]; OR ≥3 consultations 0.56, 95% CI [0.32-0.96]; OR substantial symptoms 0.36, 95% CI [0.14-0.94]). However, compared with nonrecipients, explantation patients still had higher odds of any symptom, ≥2 symptoms, and ≥3 consultations after explantation. Replacement patients also had a reduced likelihood of any symptom, ≥2 symptoms, and ≥3 consultations from pre- to postsurgery.

**Conclusions:**

Although explantation appears to improve BII-related health symptoms within a year, patients still showed an increased likelihood of symptoms compared with nonrecipients.

**Level of Evidence: 3:**

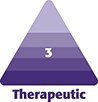

Breast implant illness (BII) comprises a constellation of nonspecific health symptoms that may emerge after the implantation of silicone breast implants. Reported symptoms span diverse organ systems, encompassing manifestations such as fatigue, hair loss, myalgia, and arthralgia.^1,2^ There are no international validated diagnostic criteria for BII, and these nonspecific health complaints are also frequently reported in the general population.^3^ Although a definitive causal link between breast implants and BII has not been established, explantation surgeries have become increasingly common worldwide, rising by 40.2% between 2019 and 2023.^4^ In the Netherlands, the number of explantations performed, specifically following concerns about BII, has also seen a notable increase recently.^5^ This trend is likely influenced by heightened media coverage of BII and previous research showing significant health improvements following explantation.^1,6-17^ However, these previous studies on explantation have generally faced several methodological limitations, including small sample sizes, selection biases because of limited settings (eg, patients treated by 1 plastic surgeon), and short follow-up periods rarely exceeding a few months. Furthermore, although these studies have shown improvements in health symptoms or stress, the follow-up assessments relied solely on self-reported data collected through surveys or telephone interviews. This may have introduced information bias, potentially leading to false or missed associations.^18^

To address these limitations, we examined health symptoms related to BII 1 year before and 1 year after explantation by linking data from a national population-based breast implant registry with general practice records. This approach allowed us to compare physician-assessed health symptoms in a representative cohort of female patients who underwent explantation with those who had breast implant replacement surgery and with patients without breast implants. Our aim was to provide a more comprehensive understanding of the impact of explantation on symptom occurrence.

## METHODS

### Data Sources

A retrospective cohort study was designed to examine the effect of explantation on health symptoms. Patients who underwent explantation or replacement surgery were identified through the nationwide Dutch Breast Implant Registry (DBIR), a population-based registry that has been prospectively recording virtually all breast implant surgeries in the Netherlands since April 2015.^19,20^ DBIR provides information on implant and surgical characteristics, as registered by the treating plastic surgeon, including the reason for explantation or replacement of the breast implant(s), such as local complications as well as BII.

This dataset was linked to Nivel Primary Care Database (Nivel-PCD) (2014-2022) and Statistics Netherlands. Nivel-PCD contains routinely recorded electronic health records data from >400 general practices across the Netherlands, representing ∼1.9 million individuals of the Dutch population.^21^ This general practice data encompasses information on consultations, referrals, comorbidities, and prescribed medications. Almost all inhabitants in the Netherlands are registered with a general practice. As a result, electronic health records contain information not only about patients who have visited their general practitioner (GP), but also about those who have not. GPs serve as gatekeepers for access to specialized care through a referral system. In the Netherlands, consultations with GPs are easily accessible because they are free of charge and supported by a comprehensive network of services. These aspects of Dutch healthcare make data from general practices well-suited for estimating prevalence rates. Moreover, these data are free from recall bias and includes professionally assessed symptoms rather than self-reported data.^22-24^ Statistics Netherlands provided data on age, household composition, migration background, degree of place of residence urbanization, household income level, and level of education. The study period for the combined dataset spanned from January 2014 to December 2022.

### Patient Selection

Patients were included in this study if they had undergone explantation surgery of cosmetic breast implants between 2016 and 2021, and had been listed with a Nivel-PCD general practice for 12 months before and until 12 months after the explantation (index date). Two control groups were selected: (1) patients who had undergone replacement surgery of cosmetic breast implants between 2016 and 2021 (replacement patients), and (2) patients without breast implants (nonrecipients), who had been enlisted in a general practice of Nivel-PCD for the same time period. Patients were classified as nonrecipients, if there was no record of breast implant surgery in DBIR (2015-2022) and Nivel-PCD (2014-2022). These control groups were selected to enable robust comparisons: 1 group consisting of patients with no exposure to breast implants, and the other serving as an active comparator. This approach accounts for potential psychological and lifestyle differences, such as smoking, that typically distinguish patients with breast implants from those without.^25^ Nonrecipients were matched with a 2:1 control-to-case ratio on age (within 5-year interval), and general practice, and were assigned the same index date as their matched explantation patient.

Inclusion criteria required that patients be ≥18 years old, have no history of breast cancer according to DBIR and Nivel-PCD, and have breast implants for at least 12 months before explantation or replacement. Patients with silicone breast implants of any type—encompassing various fillings, textures, and manufacturers—were eligible for inclusion in the study, whereas patients with tissue expanders were excluded. Explantation and replacement patients who underwent surgery because of acute postoperative complications (ie, hematoma, skin necrosis, or wound infection), confirmed breast implant-associated anaplastic large cell lymphoma, or recall reasons were excluded.

Ethics approval for this study was waived by the medical ethics committee of the University Medical Center Maastricht (reference number: 2021-2515). In the Netherlands, the use of electronic health records for research purposes is allowed under certain conditions. When these conditions are fulfilled, neither obtaining informed consent from patients nor approval by a medical ethics committee is obligatory for this type of observational studies containing no directly identifiable data (art. 24 GDPR Implementation Act jo art. 9.2 sub j GDPR). Furthermore, this study was approved according to the governance code of Nivel Primary Care Database, under number NZR-00323.005 and according to the governance code of DBIR, under number DBIR-2022-158. This report follows the STROBE reporting guideline for cohort studies.

### Outcomes

The primary outcome of this study was the occurrence of health symptoms as recorded in general practice. These symptoms were routinely recorded in electronic health records during consultations, home visits, or telephone/email consultations with a GP or practice nurse. Patients who did not consult their GP during the study period could be included in the study because of the reimbursement system, which is partly based on capitation and requires every Dutch citizen to be listed as a patient in a general practice. Multiple symptoms could be documented in every single consultation, using the International Classification for Primary Care, version 1 (ICPC).^26^ This system is used in every Dutch general practice and allows for symptom recording if diagnoses are (yet) unclear.

There is no validated set of symptoms for BII, and symptoms were selected based on Yzermans et al's study on nonspecific symptoms and previous BII studies.^1,2,6,27,28^ The present study focused on 13 symptoms: arthralgia, myalgia, nervous system symptoms, heart symptoms, gastrointestinal symptoms, mental and cognitive symptoms, skin rash, weight changes, fatigue, eye symptoms, ear symptoms, alopecia, and enlarged lymph nodes. A complete list of ICPC-coded symptoms is available in Supplemental Table 1.

The occurrence of symptoms was assessed during baseline and follow-up as any (≥1) symptom, ≥2 and ≥3 distinct symptoms, ≥3 consultations, and substantial symptoms. Substantial symptoms were defined as having 3 or more distinct symptoms, with at least 2 consultations for 2 of these symptoms within a year. These guidelines were chosen to differentiate significant health complaints from temporary symptoms, accounting for the different ways BII may present itself. The decision to focus on the number of symptoms rather than individual symptoms reflects the lack of a consistent symptom profile for BII.

### Statistical Analysis

Differences in patient characteristics across the 3 groups were assessed using χ^2^ tests for categorical variables, analysis of variance for normally distributed continuous variables, and the Wilcoxon rank-sum test for nonnormally distributed continuous variables. If the overall test indicated significant differences, we performed pairwise post hoc comparisons with Bonferroni correction to identify specific group differences.

Differences in the occurrence of symptoms between explantation patients and replacement patients, and between explantation patients and nonrecipients were examined at baseline and at follow-up with multivariable binary logistic regression. Odds ratios (ORs) were adjusted for age, general practice electronic health record system supplier, household composition, medical conditions (allergies, autoimmune diseases, mental illnesses, and cardiovascular diseases; see Supplemental Table 2 for the complete list), and medication use (allergy medication and antidepressants; Supplemental Table 3) at baseline in all comparisons. For comparisons between explantation and replacement patients, estimates were additionally adjusted for overweight status (BMI ≥25 kg/m^2^; yes/no), smoking status (yes/no), and year of surgery. Missing data on overweight and smoking status were imputed using multiple imputation by chained equations, with 22 multiple imputation datasets.^29^ The imputation model included auxiliaries age, migration background, household composition, and type of surgery (explantation/replacement).

Comparisons of symptom occurrence over time were made with multilevel mixed-effects binary logistic regression, comparing the 1-year follow-up period to the 1-year baseline period for each group of patients. The multilevel models featured a 2-level hierarchical structure: year at the first level, nested within individuals at the second level. Additionally, for symptoms that showed a significant decrease in likelihood after explantation, the percentage of patients who had the symptom during the baseline period but not during follow-up was calculated relative to those who experienced the symptom at baseline. Statistical significance was determined with a *P*-value of <.05 using 2-sided tests. All statistical analyses were performed using STATA software (version 16.1; Stata Corporation, College Station, TX).

## RESULTS

### Patient Characteristics

In total, 217 patients who underwent explantation, 228 patients who underwent replacement, and 433 patients without breast implants were included in this study (Supplemental Figure 1). The groups differed significantly in age, with mean (standard deviation [SD]) ages of 51.1 (12.3), 49.7 (11.6), and 54.4 (12.7) years, respectively (*P* < .001; Table 1). Both explantation patients and replacement patients were more likely to have a mental illness during baseline than nonrecipients. Compared with replacement patients, explantation patients were more likely to be overweight, and less likely to smoke (*P* < .05). There were no significant baseline differences between the 3 groups in terms of allergies and autoimmune diseases. Explantation was performed after a median time of 15.5 years following implantation. In the replacement group, the median time to implant replacement was 14 years. Almost all explantation patients underwent their implant removal in the most recent years between 2019 and 2021, whereas 28.9% of replacement patients underwent their replacement surgery before 2019. BII was registered as the reason for explantation in 37 patients (17.1%). The majority of explantation patients underwent either partial capsulectomy (35.5%) or full capsulectomy (40.6%), similar to replacement patients. Explantation patients more often underwent additional mastopexy during their surgery compared with replacement patients (18.0% vs 8.3%, *P* < .01). In the replacement group, nearly all patients (96.5%) opted for silicone-filled instead of saline-filled implants.

**Table 1:** General Characteristics of Study Population

	Explantation patients *n* = 217	Replacement patients *n* = 228	Patients without breast implants *n* = 433	*P*-value
	% (*n*)	% (*n*)	% (*n*)	
Demographics at index date				
Age in years, mean ± SD	51.1 ± 12.3	49.7 ± 11.6	54.4 ± 12.7	<.001^a,b^
Migration background	21.2 (46)	20.6 (47)	25.2 (109)	.32
Degree of residence urbanization				.48
Extremely urbanized	24.0 (52)	25.0 (57)	23.8 (103)	
Strongly urbanized	33.6 (73)	31.6 (72)	34.2 (148)	
Moderately urbanized	14.3 (31)	20.6 (47)	14.3 (62)	
Hardly or not urbanized	25.8 (56)	21.9 (50)	25.2 (109)	
Unknown	2.3 (5)	0.9 (2)	2.5 (11)	
Household composition				<.001^b^
Single-person household	18.4 (40)	14.9 (34)	15.5 (67)	
Couple with children	35.0 (76)	43.0 (98)	37.6 (163)	
Single parent with children	13.4 (29)	18.9 (43)	8.1 (35)	
Other	33.2 (72)	23.2 (53)	38.8 (168)	
Socioeconomic indicators at index date				
Income level				.78
Low	32.3 (70)	32.0 (73)	31.2 (135)	
Mid	39.2 (85)	38.2 (87)	43.7 (189)	
High	28.1 (61)	29.4 (67)	24.5 (106)	
Unknown	0.5 (1)	0.4 (1)	0.7 (3)	
Medical conditions in the baseline period				
Allergy	31.3 (68)	29.4 (67)	25.9 (112)	.30
Autoimmune disease	15.2 (33)	12.7 (29)	17.6 (76)	.26
Mental illness	21.7 (47)	16.2 (37)	7.6 (33)	<.001^a,b^
Cardiovascular disease	23.5 (51)	27.2 (62)	30.3 (131)	.19
Medication use in the baseline period^c^				
Allergy medication	37.3 (81)	34.7 (79)	30.5 (132)	.19
Antidepressant	17.5 (38)	14.5 (33)	8.8 (38)	.003^a^
*Characteristics of the 2 surgery groups*				
Year of surgery				<.001
2016-2018	9.4 (20)	28.9 (66)	Same index date (ie, year of surgery) as explantation patients	
2019	19.3 (41)	19.7 (45)		
2020	32.1 (68)	21.9 (50)		
2021	39.2 (83)	29.4 (67)		
Time to explantation/replacement, median (IQR)	15.5 (10.0-21.0)	14.0 (10.0-18.0)	NA	.14
Reason for explantation/replacement^d^			NA	
Breast implant illness	17.1 (37)	(<10)^e^		<.001
Capsular contracture	30.4 (66)	38.6 (88)		.07
Breast pain	18.4 (40)	15.4 (35)		.39
Dissatisfaction with volume	4.6 (10)	24.6 (56)		<.001
Asymmetry	6.9 (15)	12.3 (28)		.06
Implant rupture	23.0 (50)	29.8 (68)		.11
Silicone extravasation	13.4 (29)	17.1 (39)		.27
Other^f^	6.9 (15)	11.8 (27)		.08
Reason unknown	25.8 (56)	16.2 (37)		.01
Overweight (BMI ≥25 kg/m^2^)			NA	<.001
No	53.5 (116)	73.7 (168)		
Yes	30.4 (66)	19.3 (44)		
Unknown	16.1 (35)	7.0 (16)		
Smoking status			NA	.01
No	69.6 (151)	66.7 (152)		
Yes	10.6 (23)	19.7 (45)		
Unknown	19.8 (43)	13.6 (31)		
Filling of inserted implant during replacement	NA		NA	
Silicone		96.5 (220)		
Other		3.5 (8)		
Capsulectomy				.77
No	23.0 (50)	21.1 (48)		
Partial capsulectomy	35.5 (77)	40.4 (92)		
Full capsulectomy	40.6 (88)	37.7 (86)		
Unknown	0.9 (2)	0.9 (2)		
Mastopexy				.008
No	81.1 (176)	91.2 (208)		
Yes	18.0 (39)	8.3 (19)		
Unknown	0.9 (2)	0.4 (1)		

*P*-values calculated with χ^2^ test (categorical variables), analysis of variance (continuous variables; mean), and Wilcoxon rank-sum test (continuous variables; median). IQR, interquartile range; NA, not applicable; SD, standard deviation.

^a^Post hoc analysis showed a significant difference between explantation patients and nonrecipients.

^b^Post hoc analysis showed a significant difference between replacement patients and nonrecipients.

^c^Based on at least 1 prescription in the baseline period.

^d^Multiple reasons can be registered; consequently, percentages do not add up to 100%.

^e^Exact number of observations not reported because of disclosure risk guidelines.

^f^Implant malposition, suspicion of BIA-ALCL (not confirmed), skin scarring, flap problems, and seroma.

### Health Symptoms Before and After Surgery

**Figure 1.**
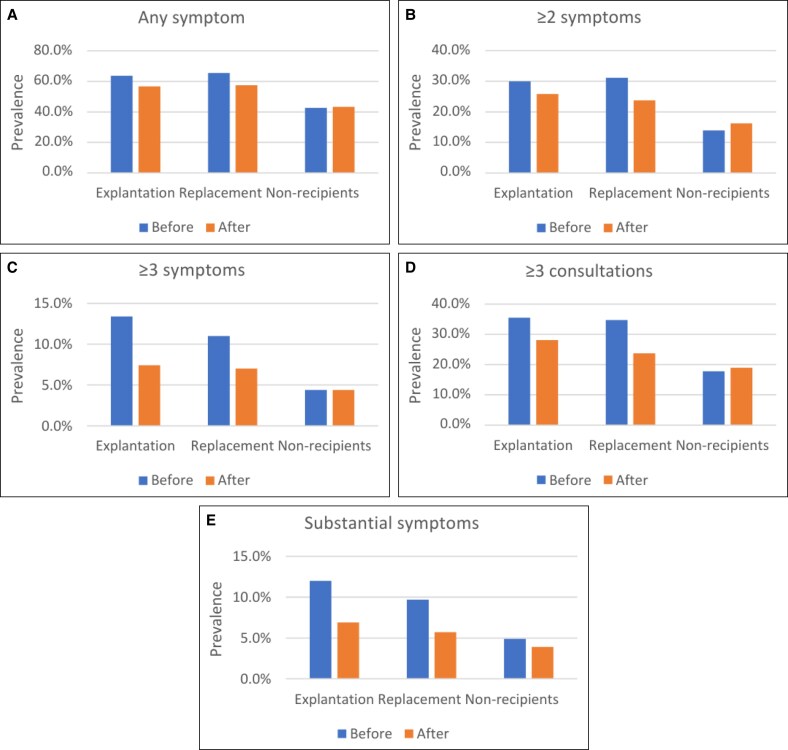
(A) Prevalence of health symptoms before and after surgery, any symptom. (B) Prevalence of health symptoms before and after surgery, ≥2 symptoms. (C) Prevalence of health symptoms before and after surgery, ≥3 symptoms. (D) Prevalence of health symptoms before and after surgery, ≥3 consultations. (E) Prevalence of health symptoms before and after surgery, substantial symptoms.

Before surgery, 63.6% of explantation patients, 65.4% of replacement patients, and 42.7% of nonrecipients had at least 1 BII-related health symptom (Figure 1). In the year following implant removal, explantation patients had a reduced likelihood of experiencing ≥3 distinct symptoms (OR 0.26; 95% CI [0.08-0.85]), having ≥3 consultations (OR 0.56; 95% CI [0.32-0.96]), and experiencing substantial symptoms (OR 0.36; 95% CI [0.14-0.94]) compared with before explantation (Table 2). In total, 65.5% and 69.2% of patients who had ≥3 distinct symptoms or substantial symptoms before explantation, respectively, no longer experienced this quantity of symptoms after implant removal (Table 3).

**Table 2:** Comparisons of Symptoms Before and 1 Year After Surgery Among Explantation Patients (*n* = 217), Replacement Patients (*n* = 228), and Nonrecipients (*n* = 433)

	1 year after surgery vs 1 year before surgery					
	Explantation patients		Replacement patients		Nonrecipients	
Symptom	OR (95% CI)	*P*-value	OR (95% CI)	*P*-value	OR (95% CI)	*P*-value
Any relevant symptom	0.66 (0.41-1.05)	.08	0.59 (0.36-0.96)	.03	1.04 (0.75-1.45)	.80
≥2 distinct symptoms	0.72 (0.42-1.23)	.23	0.60 (0.36-0.98)	.04	1.26 (0.83-1.92)	.29
≥3 distinct symptoms	0.26 (0.08-0.85)	.02	0.59 (0.30-1.17)	.13	0.74 (0.37-1.47)	.39
≥3 consultations	0.56 (0.32-0.96)	.04	0.49 (0.30-0.80)	<.01	1.14 (0.73-1.78)	.57
Substantial symptoms^a^	0.36 (0.14-0.94)	.04	0.55 (0.26-1.15)	.11	0.75 (0.36-1.59)	.45

Odds ratios (ORs) calculated with multilevel mixed-effects binary logistic regression, using a 2-level structure with year at Level 1 nested within individual patients at Level 2.

^a^≥3 distinct symptoms and for at least 2 symptoms ≥2 consultations.

**Table 3:** Changes in Symptom Occurrence After Explantation Among Explantation Patients (*n* = 217)

Symptom change	% (*n*) of patients who showed a decrease in symptom occurrence^a^
From ≥3 symptoms to <3 symptoms	65.5% (19 out of 29 patients)
From ≥3 consultations to <3 consultations	46.8% (36 out of 77 patients)
From substantial symptoms^b^ to no substantial symptoms	69.2% (18 out of 26 patients)

^a^Percentage (number) of patients who had the symptom/outcome during baseline but not during follow-up, relative to those with the symptom/outcome during baseline.

^b^≥3 distinct symptoms and for at least 2 symptoms ≥2 consultations.

Similarly, replacement patients showed a decreased likelihood of having ≥3 consultations post-replacement surgery, as well as reduced odds of any symptom and ≥2 distinct symptoms (Table 3). Nonrecipients showed no significant changes in symptom occurrence over time.

### Health Symptoms Between Groups Comparisons

Compared with nonrecipients, explantation patients had an increased likelihood of all outcomes before explantation, including ≥3 distinct symptoms (adjusted OR 2.34; 95% CI [1.27-4.34]), ≥3 consultations (adjusted OR 2.41; 95% CI [1.63-3.57], and substantial symptoms (adjusted OR 2.49; 95% CI [1.30-4.78]; Table 4). After explantation, these patients no longer had increased odds of ≥3 distinct symptoms and substantial symptoms compared with nonrecipients. However, after adjusting for confounders, explantation patients still showed an increased likelihood of any symptom, ≥2 distinct symptoms, and ≥3 consultations postsurgery compared with nonrecipients.

**Table 4:** Comparisons of Symptoms Between Explantation Patients (*n* = 217) Replacement Patients (*n* = 228), and Nonrecipients (*n* = 433) During Baseline and Follow-up

	1 year before surgery/index					1 year after surgery/index				
Symptom	Explantation patients	Replacement patients	Adjusted OR (95% CI)	Nonrecipients	Adjusted OR (95% CI)	Explantation patients	Replacement patients	Adjusted OR (95% CI)	Nonrecipients	Adjusted OR (95% CI)
Any symptom	63.6 (138)	65.4 (149)	0.92 (0.60-1.41)	42.7 (185)	2.25 (1.58-3.21)^a^	56.7 (123)	57.5 (131)	0.96 (0.63-1.45)	43.4 (188)	1.56 (1.11-2.21)^a^
≥2 distinct symptoms	30.0 (65)	31.1 (71)	1.04 (0.66-1.65)	13.9 (60)	2.43 (1.57-3.74)^a^	25.8 (56)	23.7 (54)	1.35 (0.83-2.19)	16.2 (70)	1.74 (1.14-2.65)^a^
≥3 distinct symptoms	13.4 (29)	11.0 (25)	1.37 (0.72-2.61)	5.5 (24)	2.34 (1.27-4.34)^a^	7.4 (16)	7.0 (16)	1.20 (0.52-2.81)	4.4 (19)	1.30 (0.60-2.81)
≥3 consultations	35.5 (77)	34.7 (79)	1.04 (0.67-1.60)	17.8 (77)	2.41 (1.63-3.57)^a^	28.1 (61)	23.7 (54)	1.37 (0.85-2.19)	18.9 (82)	1.65 (1.10-2.49)^a^
Substantial symptoms^b^	12.0 (26)	9.7 (22)	1.43 (0.72-2.84)	4.9 (21)	2.49 (1.30-4.78)^a^	6.9 (15)	5.7 (13)	1.58 (0.64-3.90)	3.9 (17)	1.43 (0.64-3.21)

Odds ratios (ORs) calculated with binary logistic regression, adjusted for age, general practice electronic health record system supplier, household composition, medical conditions and medical use in baseline, and additionally adjusted for overweight (yes/no), smoking status (yes/no), and year of surgery (comparisons between explantation patients and replacement patients).

^a^ *P* < .05.

^b^≥3 distinct symptoms and for at least 2 symptoms ≥2 consultations.

Compared with replacement patients, explantation patients showed no significant differences in symptom occurrence both before and after surgery.

## DISCUSSION

Because of growing concerns about BII, characterized by a range of health symptoms, more women are requesting explantation with the hope of alleviation of these symptoms.^5^ Our retrospective cohort study found a significant decrease in the likelihood of experiencing ≥3 BII-related health symptoms, ≥3 general practice consultations, and a combination of both (defined as substantial symptoms) in the year after explantation compared with before. Notably, 65.5% of patients who had ≥3 distinct symptoms before explantation, no longer experienced this quantity of symptoms afterwards.

Similar improvements in symptoms have been observed in other studies. For example, the researchers of a study found that 98% of women with typical BII symptoms before explantation experienced an average symptom improvement of 62% after implant removal.^30^ In another study, the authors reported a reduction in the mean number of symptoms from 13 to 4.5 within a year after explantation.^14^ However, the authors of previous studies relied on pre- and postoperative questionnaires, which may have heightened participants’ awareness of their symptoms and perceptions of improvement, potentially overestimating the benefits of explantation. In contrast, in this study, we are the first to examine symptoms as recorded in general practice during routine care, including both a relatively long follow-up period and a large study population. By using general practice data, we minimized the risk of bias from heightened awareness of BII, because patients were not directly questioned about implant-related health. Additionally, we focused on symptoms significant enough to prompt a visit to a GP, further reducing the likelihood of reporting less severe health issues. This methodologically rigorous approach yielded findings similar to those of previous studies and strengthens the evidence for the health benefits of explantation. Moving forward, research with follow-up periods beyond 1 year is needed, incorporating postoperative data from GPs, plastic surgeons, and specialized outpatient clinics focusing on silicone implant-related care to gain a more comprehensive understanding of the course of health symptoms over time.

Although explantation reduced the likelihood of having multiple symptoms and consultations, patients who underwent explantation remained more likely to experience 2 or more distinct symptoms and have 3 or more consultations compared with women without breast implants. This is consistent with a previous study, which found that patients who underwent explantation still reported more symptoms than those who had mastopexy without previous implants, despite significant symptom improvement after explantation.^14^ These findings suggest that although explantation improves symptoms, some issues may persist. However, it is important to note that not all patients in our study underwent explantation specifically for BII, although BII may have been underreported in DBIR. Consequently, drawing definitive conclusions about the beneficial effects of explantation on BII remains challenging.

Patients who opted for replacement surgery rather than explantation also showed a reduced likelihood of having symptoms and consultations, although the improvement appeared to be less pronounced compared with patients who underwent explantation. in a recent study, the authors similarly found that, although both replacement and explantation led to symptom improvement, explantation resulted in more substantial improvements.^17^ In another study, the researchers observed improvement in headaches following replacement surgery, whereas explantation patients reported improvements in a wider range of symptoms from 2 to 22 distinct symptoms.^10^

The observed reduction in BII-related symptoms following replacement surgery is striking, especially because replacement in this study was typically performed to address local complications or dissatisfaction with implant size, rather than to treat BII. It is possible that the symptom improvement partly reflects a placebo effect, driven by positive treatment expectations.^31^ Nearly 30% of replacements were because of ruptured implants, and patients may anticipate symptom relief following the removal of a ruptured implant. Although evidence increasingly suggests that silicone leakage is unlikely to be the cause of symptoms,^32,33^ its portrayal in the media as a likely contributor may shape these expectations. Similarly, in the case of explantation, symptom relief may also result partially or entirely from a placebo effect, influenced by (social) media reports that highlight the health benefits of implant removal. This is especially evident as most explantation procedures occurred between 2019 and 2021, coinciding with heightened media attention on BII in the Netherlands. This media focus may have influenced the rise in explantations and reinforced placebo effects.

In cases of implant replacement because of dissatisfaction or asymmetry, aesthetic improvements with replacement may enhance the psychosocial well-being and self-esteem, similar to the effects seen with primary augmentation.^34^ Because psychological well-being has been linked to physical health,^35^ this could potentially contribute to the improvement of BII-like symptoms.

Overall, the exact cause of health symptom improvement after replacement surgery remains unclear, underscoring the need for further research to explore the mechanisms behind symptom improvement following both explantation and replacement procedures.

Multiple potential underlying mechanisms of BII are discussed in the literature.^36^ One proposed mechanism for BII is related to psychological factors and somatoform symptom disorder. Notably, both explantation and replacement patients displayed higher rates of mental illness compared with women without breast implants, with 1 in 5 patients in the explantation group affected. Our previous research also found a higher prevalence of mental illness among patients before cosmetic breast augmentation using breast implants compared with those who did not undergo this procedure.^37^ Furthermore, previous research has shown that patients with breast implants who have systemic symptoms they attribute to their implants experience higher levels of anxiety compared with those without such symptoms.^14^ Additionally, explantation patients with self-reported BII symptoms have a higher prevalence of preexisting anxiety and depression disorders before breast implantation compared with other breast (implant) surgery groups without symptoms.^38^ Moreover, in another study, the authors found that patients with cosmetic breast implants exhibited higher levels of neuroticism compared with normative data.^39^ Neuroticism is a personality trait associated with emotional responses to frustrations, losses, or threats. Individuals with high levels of neuroticism often experience more intense emotional reactions and a greater frequency of somatic symptoms, as well as increased healthcare utilization.^40,41^ It could be hypothesized that neuroticism may also interfere with the resolution or persistence of symptoms following implant removal.

Another potential mechanism suggests that BII symptoms may arise from an immune system hyperreaction to breast implants.^36^ The authors of previous research have reported high rates of allergies and autoimmune diseases among patients with BII symptoms.^1,6,15,17,42,43^ Interestingly, in our study, we found no significant difference in the prevalence of allergies or autoimmune diseases across the 3 groups of patients. The evidence linking breast implants to autoimmune diseases remains inconclusive;^44^ whereas in some studies, the authors have reported an increased risk of connective tissue diseases in patients with breast implants,^45,46^ others did not find a significant association.^47,48^ Lee et al^49^ reached similar conclusions, noting no substantial increase in risk but pointing out the challenges in studying autoimmune diseases in patients with breast implants, such as difficulties in disease ascertainment and accessing medical records for self-reported cases. In our study, information on autoimmune diseases was extracted from the general practice database for all patients, minimizing the likelihood of undiagnosed cases affecting 1 group more than another. This is further supported by the Dutch healthcare system, where the GP serves as the gatekeeper to specialized care and visits are free of charge, ensuring equal access for all residents. Thus, our findings provide reliable estimates of autoimmune disease prevalence in both females undergoing explantation and those without breast implants and seem to challenge the autoimmune disease hypothesis.

### Strengths and Limitations

The strengths of our study include the relatively large study population, the inclusion of multiple control groups, comprehensive confounder adjustment, and physician-verified symptoms. Additionally, patient selection was based on nationwide breast implant registry data, minimizing the selection bias often seen in single-center or single-surgeon explantation studies. By including all cosmetic breast implant explantations, our study provides a broader perspective on health symptoms experienced by patients undergoing explantation, rather than focusing only on cases with BII as the registered reason, which can be influenced by individual surgeons’ knowledge and beliefs.

Our study also has some limitations. Although we examined a broad range of symptoms, general practice data may not capture all health issues or the impact of these symptoms on daily life. Persistent health symptoms may be discussed with the patient's plastic surgeon rather than her GP after implant removal. Therefore, future studies would benefit from integrating GP data with postoperative records from plastic surgeons to provide a more comprehensive understanding of the course of health outcomes over time. Although our 1-year baseline and follow-up periods exceed those of most previous studies, longer follow-up is needed to assess long-term effects. Moreover, small subgroup sizes and disclosure risk guidelines for routinely recorded data limited our ability to publish detailed patient characteristics with few observations. As a result, we could not report the prevalence of specific mental illnesses or perform subanalyses in patients who underwent explantation because of BII. As the DBIR database expands, future research should explore differences in characteristics, including psychological profiles, between explantation patients with and without BII to better understand who may be more susceptible to it. Additionally, the lack of data on smoking and BMI for nonrecipients limited our ability to adjust for these confounders in comparisons between explantation patients and nonrecipients.

## CONCLUSIONS

In this study, we found that female patients who underwent explantation of cosmetic breast implants had a reduced likelihood of having multiple BII-related health symptoms and general practice consultations in the year following the procedure. However, even after implant removal, these patients remained more likely to experience health symptoms and have multiple GP consultations for BII-related symptoms compared with women without breast implants. Additionally, patients who opted for breast implant replacement surgery also showed a reduced likelihood of symptoms and consultations. The extent to which these symptoms are causally linked to the breast implants remains uncertain and could not be determined in this study. Future research should explore the underlying mechanisms and investigate the long-term effects of both explantation and breast implant replacement.

## Supplemental Material

This article contains supplemental material located online at https://doi.org/10.1093/asj/sjaf030.

## Supplementary Material

sjaf030_Supplementary_Data
