# Supplementary material for: The Occurrence of Health Symptoms in General Practice Before and After the Explantation of Cosmetic Breast Implants
Source: Aesthet Surg J. 2025 Feb 19;45(6):589–98. doi: 10.1093/asj/sjaf030 (PMC12080887; doi:10.1093/asj/sjaf030)
Supplement: sjaf030_Supplementary_Data [file sjaf030_supplementary_data.zip › Supplemental Digital Content 1.docx]

| Supplemental Digital Content 1 Table. Definitions of symptoms | | |
| --- | --- | --- |
| Symptom | ICPC | Description |
| Arthralgia | L09 | Arm symptom/complaint |
|  | L10 | Elbow symptom/complaint |
|  | L11 | Wrist symptom/complaint |
|  | L12 | Hand/finger symptom/complaint |
|  | L13 | Hip symptom/complaint |
|  | L14 | Leg/thigh symptom/complaint |
|  | L15 | Knee symptom/complaint |
|  | L17 | Foot/toe symptom/complaint |
|  | L01 | Neck symptom/complaint |
|  | L08 | Shoulder symptom/complaint |
|  | L02 | Back symptom/complaint |
|  | L03 | Low back symptom/complaint |
|  | L20 | Joint symptom/complaint NOS |
| Myalgia | L18 | Muscle pain |
| Nervous system complaints | N17 | Vertigo |
|  | N01 | Headache |
|  | N02 | Tension headache |
|  | N05 | Tingling fingers/feet/toes |
| Heart complaints | K04 | Palpitations/awareness of heart |
|  | L04 | Chest symptom/complaint |
|  | K01 | Heart pain |
|  | K02 | Pressure/tightness of heart |
|  | K03 | Cardiovascular pain NOS |
| Gastrointestinal complaints | D01 | Abdominal pain/cramps general |
|  | D02 | Abdominal pain epigastric |
|  | D06 | Abdominal pain localized other |
|  | D11 | Diarrhoea |
|  | D12 | Constipation |
|  | D09 | Nausea |
| Weight changes | T07 | Weight gain |
|  | T08 | Weight loss |
| Fatigue | A04 | Fatigue/tiredness general |
| Mental/cognitive complaints | P01 | Feeling anxious/nervous/tense |
|  | P02 | Acute stress reaction |
|  | P03 | Feeling depressed |
|  | P04 | Feeling/behaving irritable/angry |
|  | P06 | Sleep disturbance |
|  | P20 | Memory disturbance |
| Skin rash | S01 | Pain/tenderness of skin |
|  | S06 | Rash localized |
|  | S07 | Rash generalized |
|  | S02 | Pruritus |
|  | S88 | Dermatitis contact/allergic |
| Eye complaints | F01 | Eye pain |
|  | F02 | Red eye |
|  | F99 | Eye/adnexa disease other (including insufficiency of tear film) |
| Ear complaints | H01 | Ear pain/earache |
|  | H02 | Hearing complaint |
|  | H03 | Tinnitus, ringing/buzzing ear |
|  | H13 | Plugged feeling ear |
| Alopecia | S23 | Hair loss/baldness |
| Enlarged lymph node(s) | B02 | Lymph gland(s) enlarged/painful |
|  | B03 | Lymph gland(s) symptom/complaint other |
