# Supplementary material for: The Occurrence of Health Symptoms in General Practice Before and After the Explantation of Cosmetic Breast Implants
Source: Aesthet Surg J. 2025 Feb 19;45(6):589–98. doi: 10.1093/asj/sjaf030 (PMC12080887; doi:10.1093/asj/sjaf030)
Supplement: sjaf030_Supplementary_Data [file sjaf030_supplementary_data.zip › Supplemental Digital Content 2.docx]

| Supplemental Digital Content 2 Table. List of included comorbidities | | |
| --- | --- | --- |
| Disease | ICPC | Description |
| Allergy | R96 | Asthma |
|  | R97 | Allergic rhinitis |
|  | S87 | Dermatitis/atopic eczema |
|  | A12 | Allergy/allergic reaction |
| Autoimmune disease | N86 | Multiple sclerosis |
|  | T86 | Hypothyroidism/myxoedema |
|  | T81 | Goitre |
|  | S91 | Psoriasis |
|  | D94 | Chronic enteritis/ulcerative colitis |
|  | T99 | Endocrine/metabolic/nutritional disease other |
|  | N94 | Peripheral neuritis/neuropathy |
|  | T85 | Hyperthyroidism/thyrotoxicosis |
|  | D99 | Disease digestive system other |
| Mental illness | P76 | Depressive disorder |
|  | P75 | Somatization disorder |
|  | P74 | Anxiety disorder/anxiety state |
|  | P78 | Neuraesthenia/surmenage |
|  | Z29 | Social problem NOS |
| Cancer | T71 | Malignant neoplasm thyroid |
|  | D74 | Malignant neoplasm stomach |
|  | D76 | Malignant neoplasm pancreas |
|  | U76 | Malignant neoplasm of bladder |
|  | A79 | Malignancy NOS |
|  | D75 | Malignant neoplasm colon/rectum |
|  | W72 | Malignant neoplasm related to pregnancy |
|  | U77 | Malignant neoplasm urinary other |
|  | U75 | Malignant neoplasm of kidney |
|  | X75 | Malignant neoplasm cervix |
|  | R84 | Malignant neoplasm bronchus/lung |
|  | B74 | Malignant neoplasm blood other |
|  | N74 | Malignant neoplasm nervous system |
|  | R85 | Malignant neoplasm respiratory other |
|  | S77 | Malignant neoplasm of skin |
|  | X77 | Malignant neoplasm genital female other |
|  | D77 | Malignant digestive neoplasm, other/NOS |
| Cardiovascular disease | K70 | Infection of circulatory system |
|  | K71 | Rheumatic fever/heart disease |
|  | K72 | Neoplasm cardiovascular |
|  | K73 | Congenital anomaly cardiovascular |
|  | K74 | Ischaemic heart disease with angina |
|  | K75 | Acute myocardial infarction |
|  | K76 | Ischaemic heart disease without angina |
|  | K77 | Heart failure |
|  | K78 | Atrial fibrillation/flutter |
|  | K79 | Paroxysmal tachycardia |
|  | K80 | Cardiac arrhythmia NOS |
|  | K81 | Heart/arterial murmur NOS |
|  | K82 | Pulmonary heart disease |
|  | K83 | Heart valve disease NOS |
|  | K84 | Heart disease other |
|  | K85 | Elevated blood pressure |
|  | K86 | Hypertension uncomplicated |
|  | K87 | Hypertension complicated |
|  | K88 | Postural hypotension |
|  | K89 | Transient cerebral ischaemia |
|  | K90 | Stroke/cerebrovascular accident |
|  | K91 | Cerebrovascular disease |
|  | K92 | Atherosclerosis/peripheral vascular disease |
|  | K93 | Pulmonary embolism |
|  | K94 | Phlebitis/thrombophlebitis |
|  | K95 | Varicose veins of leg |
|  | K96 | Haemorrhoids |
|  | K99 | Cardiovascular disease other |
