# Supplementary material for: The Occurrence of Health Symptoms in General Practice Before and After the Explantation of Cosmetic Breast Implants
Source: Aesthet Surg J. 2025 Feb 19;45(6):589–98. doi: 10.1093/asj/sjaf030 (PMC12080887; doi:10.1093/asj/sjaf030)
Supplement: sjaf030_Supplementary_Data [file sjaf030_supplementary_data.zip › Supplemental Digital Content 3.docx]

| Supplemental Digital Content 3 Table. List of included medications | | |
| --- | --- | --- |
| Medication | ATC | Description |
| Allergy medication | R03 | Drugs for obstructive airway diseases |
|  | R01AC | Nasal preparation of antiallergic agents |
|  | R01AD | Nasal preparation of corticosteroids |
|  | R06 | Antihistamines for systemic use |
|  | D07 | Dermatological corticosteroids |
| Antidepressants | N06A | Antidepressants |
