# Supplementary figures and images for: The Occurrence of Health Symptoms in General Practice Before and After the Explantation of Cosmetic Breast Implants
Source: Aesthet Surg J. 2025 Feb 19;45(6):589–98. doi: 10.1093/asj/sjaf030 (PMC12080887; doi:10.1093/asj/sjaf030)

Supplemental Digital Content 4 – flow diagram of study population selection

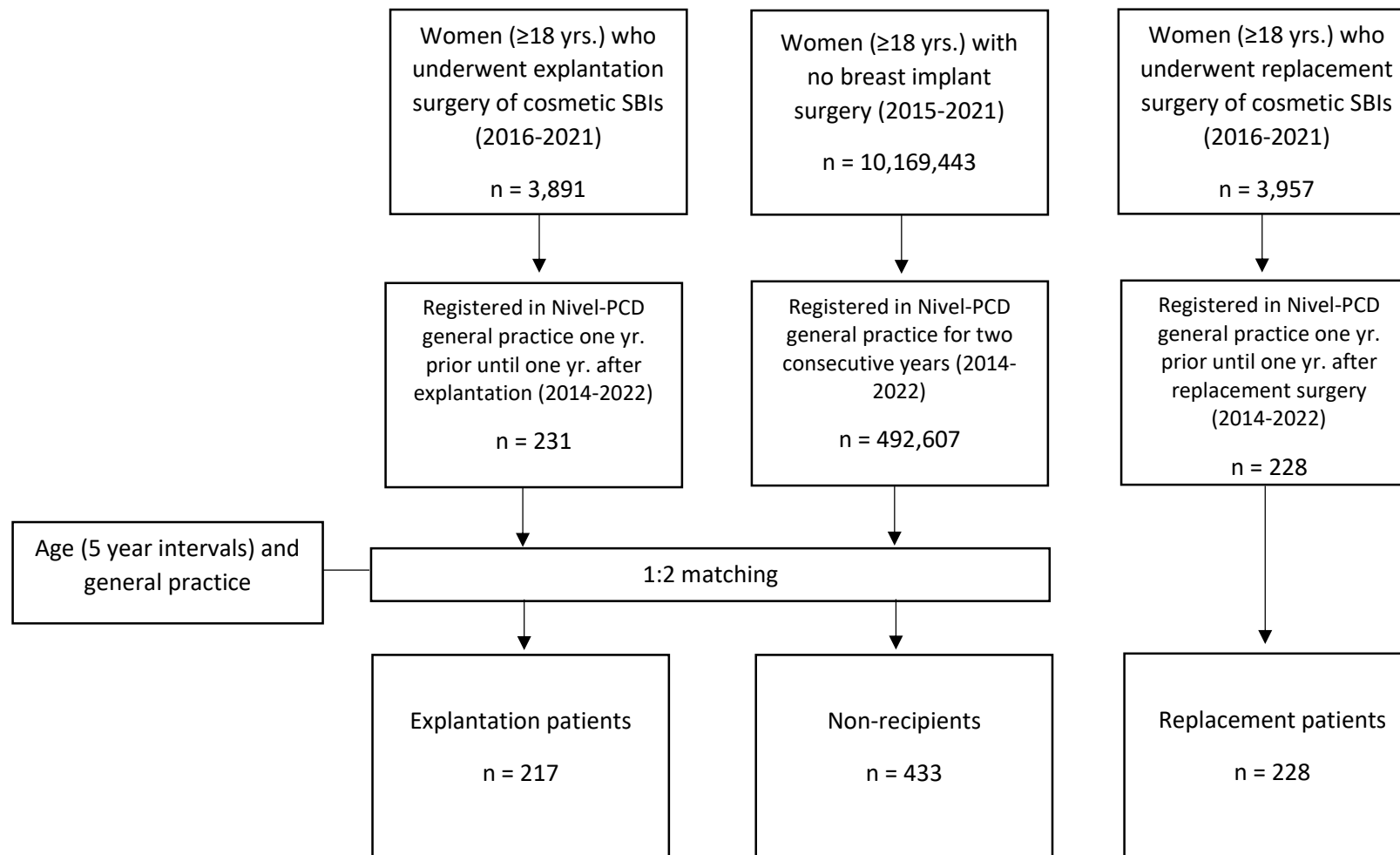

Supplement: sjaf030_Supplementary_Data [file sjaf030_supplementary_data.zip › Supplemental Digital Content 4.pdf]
